# Acne Vulgaris and Metabolic Syndrome: A Possible Association

**DOI:** 10.7759/cureus.24750

**Published:** 2022-05-05

**Authors:** Sejal Chandak, Adarshlata Singh, Bhushan Madke, Sugat Jawade, Rachit Khandelwal

**Affiliations:** 1 Dermatology, Venereology and Leprosy, Jawaharlal Nehru Medical College, Acharya Vinobha Bhave Rural Hospital (AVBRH), Wardha, IND; 2 Radiology, Dr. D. Y. Patil Medical College, Research Centre and Hospital, Pune, IND

**Keywords:** fasting blood sugar, deranged lipid profile, arterial blood pressure, diabetes and metabolic syndrome, acne vulgaris. epidemiology. life style

## Abstract

Introduction

Acne vulgaris is an androgen-dependent disorder with excessive sebum production and proliferation of *Propionibacterium acnes. *Metabolic syndrome (MetS) is a multisystem disorder that increases the risk of diabetes mellitus, stroke, and cardiovascular diseases. ​This study aims to analyze the association of MetS with acne vulgaris.

Methods

A cross-sectional study was conducted with 65 cases of acne vulgaris and 65 age and sex-matched controls. We used the system provided by the Indian authors for grading acne according to the clinical severity. In addition, the criteria updated according to the joint consensus of 2009 were employed for the diagnosis of MetS.

Results

On clinical examination, grade 2 was the most prevalent grade of acne. We observed an increased incidence of abnormal waist circumference, triglyceride, HDL, and fasting blood glucose among the cases (p<0.05). Consequently, an increased occurrence of MetS was observed in the case group (p=0.011). While comparing the mean values of the parameters, we noted a significant difference in terms of waist circumference and HDL values. An increased mean value of waist circumference was noted in the case group while an increased mean value of HDL was reported from the control group (p<0.05).

Conclusion

Patients with acne vulgaris have a greater chance of developing MetS. Hence, an in-depth examination of clinical, anthropometric, and biochemical parameters that may lead to the development of MetS is necessary.

## Introduction

Acne vulgaris is considered one of the most common skin disorders worldwide, involving chronic inflammatory infection of the pilosebaceous unit of the skin [1]. Acne affects more than 85% of adolescents and young adults [2]. Therefore, it is a vital issue during dermatological consultations because of its prevalence and impact on patients' social lives.

The first step in the pathophysiology of acne is increased sebum production, which causes follicular hyperkeratinization. This is followed by an infestation of *Propionibacterium acnes* (recently renamed *Cutibacterium acnes*), which causes the eventual release of inflammatory mediators. The usual inflammatory acne prevalent in the pubertal age group commonly occurs due to increased circulating androgen levels. Studies have also shown that an increased insulin level can aggravate acne [3].

The role of lipid metabolism and hormonal action in the differentiation of sebocytes are causative factors for acne. Insulin-like growth factor-1 (IGF-1) has also been shown to cause excess sebum production and cause acne independently [4]. A previous study reported elevated IGF-1 levels in cases of acne, potentially indicating a possible influence of insulin and growth hormone levels [5,6].

The metabolic syndrome (MetS), first described as Syndrome X by Reaven [7], comprises a set of laboratory and physical parameters predisposing the cases to the causation of cardiovascular diseases and diabetes mellitus (Type 2) [8]. Central obesity is considered one of the major constituents of MetS. The International Diabetes Federation (IDF) guideline regards it as a defining criterion. Subsequent consensus by the American Heart Association (AHA) and the National Heart, Lung, and Blood Institute (NHLBI) in 2009 recommended considering central obesity as just one of the criteria. Also, it recommended incorporating ethnicity-specific waist circumference (separately for males and females) [9].

The other parameters include elevated triglyceride levels, reduced high-density lipoproteins, elevated blood pressure, and increased fasting blood sugar. The presence of any three out of the five parameters in an individual is labeled as MetS. The pathophysiology of MetS is intricately associated with insulin resistance. The tissues like muscles, fat, and other cells, become insensitive to insulin levels in the bloodstream and fail to absorb blood glucose [10]. Central obesity and adipose tissue accumulation play a significant role in developing insulin resistance.

MetS and the skin

Systemic metabolic derangements can often result in cutaneous manifestations and vice versa [11]. The deposition of excess adipose tissue and insulin resistance in MetS initiates a spectrum of hormonal disturbances [12]. In the pathogenesis of MetS and acne, inflammatory markers like TNF-α, IL-17, IL-23, and oxidative stress have shown a possible correlation [13]. With this study, we aim to analyze the changes in markers of MetS observed in patients with acne vulgaris in contrast to those with no such skin manifestations. Identifying such a positive association between acne vulgaris and MetS at an earlier stage would enable us to take necessary preventive measures to minimize the brunt of the disease.

## Materials and methods

The study was carried out in a tertiary care hospital in Central India over two years. Institutional Ethics Committee (IEC), Datta Meghe Institute of Medical Sciences, and Jawaharlal Nehru Medical College, clearance was obtained before starting the study. The research approval number is DMIMS(DU)/IEC/Aug-2019/8229. This study was a prospective, cross-sectional, case-control study. The sample size was calculated using the formula n = [Z_α/2_]^2^ * P(1-P)/ d^2^ considering 95% confidence interval (n = sample size, Z_α/2 _= level of significance, P = prevalence and d=desired error of margin). A total of 65 clinically diagnosed cases of acne vulgaris and 65 age and sex-matched controls were included. 

Ages <18 years and >40 years, presence of any systemic comorbidities like diabetes mellitus, hypertension, dyslipidemia, etc., patients having a history of topical application of or ingestion of oral-systemic drugs (steroids, isotretinoin, etc.) for the last six weeks and dermatological disorders having any association with MetS will not be included (psoriasis, rosacea, hidradenitis suppurativa, alopecia, systemic lupus erythematosus, atopic dermatitis, etc.). Written informed consent was taken from all cases and controls of the study in their vernacular language for voluntary participation.

The same dermatologist made the diagnosis of acne vulgaris in all cases. Along with routine history, detailed history of all the participants (cases and controls) regarding the duration of acne and family history of acne was taken. In addition, the demographic data, general examination, cutaneous examination, and subsequent anthropometric and biochemical analysis were done for all cases and control group subjects. For clinical grading of acne, this study considered the Indian grading system provided by the Indian authors [14].

Height, weight, and BMI

Height measurements were recorded in centimeters using a wall-mounted scale. The individuals were made to stand erect against the wall and face forward. They were also asked to be barefoot with their feet lying flat on the floor. Weight was recorded in kilograms on a weighing scale. BMI was calculated as weight in kilograms divided by height in meters squared (kg/m^2^). The individuals were categorized as normal (18.5-24.9 kg/m^2^), overweight (25-29.9 kg/m^2^), and obese (>30 kg/m^2^). 

Waist circumference

The waist circumference was measured using a measuring tape in a standing position, halfway between the level of the lower margin of the last palpable rib superiorly and the tip of the iliac crest inferiorly.

Blood pressure

Blood pressure (mmHg) was measured at the right brachial artery using a standard sphygmomanometer cuff in a sitting position, twice in each subject, to obtain the mean value.

Laboratory investigations

The blood samples for fasting lipid profile and fasting blood sugar were taken from the anterior cubital vein, in the morning hours, after overnight fasting of 10-12 hours. The lipid profile, which includes total cholesterol, high-density lipoprotein (HDL), and triglycerides (TG), was measured.

The diagnosis of MetS was based on the Joint Interim Statement of the International Diabetes Federation Task Force on Epidemiology and Prevention; National Heart, Lung, and Blood Institute; American Heart Association; World Heart Federation; International Atherosclerosis Society; and International Association for the Study of Obesity [9]. Here, five parameters were considered, with the presence of any three confirming the diagnosis of MetS (Table 1).

**Table 1:** Revised NCEP: ATP III Criteria for Metabolic Syndrome. In accordance with the consensus statement of the International Diabetes Federation Task Force on Epidemiology and Prevention; National Heart, Lung, and Blood Institute; American Heart Association; World Heart Federation; International Atherosclerosis Society; and International Association for the Study of Obesity in 2009 [9].

Parameters	Cut-off Values
Elevated Waist Circumference (Asian population)	Men: ≥90cm
	Women: ≥80cm
Elevated Blood Pressure	Systolic Blood Pressure: ≥130 mmHg and/or
	Diastolic Blood Pressure: ≥85mmHg
Elevated Triglycerides	≥150 mg/dL (1.7 mmol/dL)
Reduced High-Density Lipoprotein	Men: <40 mg/dL (1.0 mmol/L)
	Women: <50 mg/dL (1.3 mmol/L)
Elevated Fasting Glucose	≥100mg/dL

Statistical analysis

Data were entered in a Microsoft Excel spreadsheet. All entries were double entered, checking consistency. All the analyses were performed using SPSS ver. 26.0 (IBM Corp, USA), and the statistical significance was evaluated at a 5% level. Pearson’s chi-square test was used for nonparametric data, whereas ANOVA and t-tests were applied for parametric data.

## Results

We included 65 cases of acne vulgaris and 65 age- and gender-matched controls in this study. The mean age of the case group was 23.43 ± 3.99 years, and a comparable mean age of 23.63 ± 4.03 years was observed in the control group. After clinical examination of acne lesions, we observed that out of 65 patients, 26 (40%) had grade 2 acne, followed by 24 (36.9%) with grade 1, 11 (16.9%) with grade 3, and four (6.2%) with grade 4.

There was no significant difference in both the groups concerning BMI. However, we did find a statistically significant difference in waist circumference between the two groups (p=0.003). In the control group, there were 61 (93.8%) subjects with systolic BP <130 mmHg, while four (6.2%) had ≥130 mmHg. In the case group, there were 59 (90.8%) with systolic BP <130 mmHg, while six (9.2%) had ≥130 mmHg. The difference in the distribution was not significant (p=0.510). The distribution of subjects for diastolic BP was the same in both the groups, with 59 (90.8%) subjects having <85 mmHg and six (9.2%) having ≥85 mmHg (p=0.999). When fasting blood sugar was considered, a larger number of subjects had values above the reference in the case group compared to the control group (p=0.007). Similarly, among lipid profile parameters, the difference in the distribution of subjects in the two groups regarding HDL-C (p=0.0003) and triglyceride levels (p=0.024) was significant, with increased occurrence of abnormal levels noted in the case group (Table 2).

**Table 2:** Distribution of subjects in the case and control groups according to different parameters relating to metabolic syndrome. BMI: Body mass index, SBP: Systolic blood pressure, DBP: Diastolic blood pressure, HDL-C: High-density lipoprotein-cholesterol (S): Significant

Parameters		Cases (n)	Controls (n)	P value
BMI (kg/m^2^)	Normal	41 (63.1%)	48 (73.8%)	0.326
	Overweight	18 (27.7%)	11 (27.7%)	
	Obese	6 (9.2%)	6 (9.2%)	
Waist circumference (cm)	Normal	40 (61.5%)	55 (84.6%)	0.03 (S)
	Abnormal	25 (38.5%)	10 (15.4%)	
SBP (mmHg)	Normal	59 (90.8%)	61 (93.8%)	0.51
	Abnormal	6 (9.2%)	4 (6.2%)	
DBP (mmHg)	Normal	59 (90.8%)	59 (90.8%)	0.999
	Abnormal	6 (9.2%)	6 (9.2%)	
FBS (mg/dl)	Normal	47 (72.3%)	59 (90.8%)	0.007 (S)
	Abnormal	18 (27.7%)	6 (9.2%)	
HDL-C (mg/dl)	Normal	29 (44.6%)	49 (75.4%)	0.0003 (S)
	Abnormal	36 (55.4%)	16 (24.6%)	
Triglycerides (mg/dl)	Normal	48 (73.8%)	58 (89.2%)	0.024 (S)
	Abnormal	17 (26.2%)	7 (10.8%)	
Metabolic Syndrome (%)	Yes	17 (26.2%)	6 (9.2%)	0.011 (S)

For the assessment of MetS, we used the NCEP: ATP III criteria modified in 2009. In the control group, there were six (9.2%) subjects with MetS, while in the case group, there were 17 (26.2%) subjects with the syndrome revealing a significant difference between the two groups (p=0.011) (Table 2).

The mean values for FBS and TG parameters were comparable between the two groups (p>0.05). The mean HDL level in the case group observed was 44.11 ± 7.42 mg/dL, which was significantly smaller than the control group, 48.25 ± 7.81 mg/dL (p=0.002). Whereas an elevated mean value of waist circumference was noted in the case group (p=0.0037). The mean systolic and diastolic BP were fairly similar in the two groups (Table 3).

**Table 3:** Comparison of parameters for metabolic syndrome between the two groups. HDL-C: High-density lipoprotein-cholesterol, BP: Blood Pressure, (S): Significant

Parameter	Group	N	Mean	Standard Deviation	P-value*
Fasting Blood Sugar (mg/dL)	Control	65	93.91	10.70	0.895
	Case	65	94.18	12.52	
Triglycerides (mg/dL)	Control	65	112.29	32.37	0.157
	Case	65	121.60	41.52	
HDL-C (mg/dL)	Control	65	48.25	7.81	0.002 (S)
	Case	65	44.11	7.42	
Waist Circumference (cm)	Control	65	78.14	8.97	0.037 (S)
	Case	65	81.57	9.54	
Systolic BP (mmHg)	Control	65	117.38	8.67	0.528
	Case	65	118.45	10.37	
Diastolic BP (mmHg)	Control	65	78.29	5.58	0.61

## Discussion

Our cross-sectional case-control study included a total of 130 subjects consisting of 65 cases of acne and 65 age and gender-matched controls. Acne is the most common facial disorder in the adolescent age group. It affects about 85% of young adults of both genders. The mean age for the cases with acne in our study was found to be 23.43 ± 3.99 years, which was similar to the observations of Del Prete et al., Nagpal et al., and Podder et al. who reported the mean age of 18.6 ± 2.5 years, 22.7 ± 0.6 years and 21 ± 4.9 years, respectively from their studies on acne vulgaris [15-17].

Acne vulgaris has been known to affect females more than males [18,19]. The demographic details of the acne group of our study revealed a female preponderance (61.5% females and 38.5% males). Balta et al. and Podder et al. have also observed similar genetic predispositions in their studies [17,20]. All the cases of acne were graded according to the system provided by the Indian authors [14]. In our study, the maximum cases were of grade 2 (40%), while the least were of grade 3 and grade 4 (6.2%). Furthermore, a study performed by da Cunha et al. on 416 patients of acne vulgaris also reported grade 2 to be the most prevalent [21].

We observed a similar distribution of subjects concerning BMI and blood pressure indices in the two groups. However, we noted a significantly higher proportion of individuals with abnormal waist circumference, fasting blood glucose, HDL-C levels, and triglyceride levels in the case group. The study performed by Biagi et al. reported no significant difference in the number of individuals with values of triglycerides, LDL, and HDL above the reference values in subjects with and without acne [22].

Our study reports an increased incidence of MetS in the case group consisting of patients with acne (26.2%) compared with their normal counterparts (9.2%). This difference was statistically significant (p=0.011), indicating a positive association between acne in the occurrence of MetS. Del Prete et al. reported a similar significant association in their case-control study on 22 cases of acne and their age and gender-matched controls [16]. A higher prevalence of MetS in acne patients of 32% was also reported by Podder et al.; however, the difference in comparison to the control group in their study was not significant (p=0.06) [17]. Nagpal et al. also observed a higher proportion of acne cases having MetS. In their study, 17% of subjects fulfilled MetS criteria in the acne group compared to 9% from the control group (p=0.09) [15].

When comparing the mean values of different parameters associated with MetS, we observed reasonably comparable values for SBP, DBP, and fasting blood glucose levels between the two groups. In agreement with our study, similar values of SBP and DBP between cases and controls were also reported by Podder et al. [17]. However, a higher mean value of SBP and DBP was noted in patients with acne in other studies [15,16].

In contrast to our study, Podder et al., Nagpal et al., and Del Prete et al. detected significantly increased fasting blood glucose values in the case group [15-17]. Balta et al. had a similar observation of no significant difference with respect to fasting blood glucose in the case and control groups [20]. Raised blood glucose values are associated with acne patients principally because an increase in blood glucose levels triggers insulin secretion, which decreases the binding protein for IGF-1, promoting cell proliferation by IGF-1. Higher fasting and postprandial insulin values can cause acne flare-ups by increasing basal keratinocyte proliferation. Insulin also causes stimulation of androgen secretion, ultimately leading to increased sebum production [20]. Insulin sensitivity decreases during puberty and adolescence, along with an increase in IGF-1 and insulin serum levels. Sex hormone-binding globulin (SHBG) and insulin-like growth factor-binding protein-1 (IGFBP-1) serum levels show a reduction. Insulin and IGF-1 levels, for instance, peak in late adolescence and steadily fall until the third decade. Acne appears at about the same time as the preadolescent increase in plasma insulin, IGF-1, and BMI and usually clears up by the end of puberty, even though circulating androgens stay unchanged [23].

The case group revealed an increased mean value of waist circumference in the acne patients (p=0.037). This finding was reciprocated in the study performed by Del Prete et al. [16]. However, no significant difference in waist circumference was observed in previous studies [15,17,20]. A peculiar finding of the analysis performed by Snast et al. revealed that obesity had a protective effect against acne [24]. Increased aromatase activity is seen in obese individuals, escalating the peripheral conversion of androgens to estrogens in the adipose tissue. Estrogen, having an action opposite to androgens, decreases sebum production. Furthermore, obesity also suppresses the 5- alpha-reductase 2 activity that converts testosterone to dihydrotestosterone (DHT). DHT is an essential component in the pathogenesis of acne.

Regarding lipid parameters, no statistical difference was reported in the mean value of triglycerides between the two groups (p=0.157). However, significantly increased mean values of HDL-C were recorded from the control group (p=0.002). Previous studies have also reported similar triglyceride levels in cases and controls in agreement with our study [15-17,20,22]. Similarly, an elevated HDL-C level amongst the normal control group when compared with acne cases has also been reported by most authors [15,25,26]. Studies performed by Nagpal et al. and Balta et al. did not reveal any significant difference in HDL levels in the two groups [15,20].

A study performed by Utami et al. in 2019 conferred that there are three main types of blood lipids in our body: triglycerides, phospholipids, and cholesterol [27]. De novo synthesis of blood lipids or sebaceous glands results in the formation of sebum lipids. Hence, the levels of blood lipids in the body may play a dynamic role in determining the sebum lipid composition. Follicular infundibulum epithelial hyperplasia and hyperkeratosis lead to comedone formation when lipid peroxide increases. They also cause sebaceous gland proliferation. Peroxisome proliferator-activated receptors (PPARs) play a role in lipid control, lipoprotein metabolism, inflammatory response, epidermal cell proliferation, differentiation, and sebaceous gland cell apoptosis triggered by lipid peroxides [27].

Our study reveals a positive association with MetS in patients with acne vulgaris, likely due to the biochemical and hormonal alterations expected in acne pathophysiology. However, the study's primary limitations are limited sample size and a non-uniform distribution of cases across different grades. Hence, this study could not ascertain the correlation of MetS with the increasing severity of acne. Moreover, the analysis of serum insulin levels to confirm insulin resistance was beyond the scope of the study. Therefore, a large sample size longitudinal study with a significant number of cases of each severity and an in-depth laboratory workup is recommended.

## Conclusions

Acne vulgaris is considered one of the most common skin disorders in the adolescent age group. A higher incidence of altered waist circumference, triglyceride levels, HDL levels, and fasting blood glucose levels is observed in the cases with acne compared to their normal counterparts. Consequently, a significant association between MetS with acne was observed. The association between acne vulgaris and MetS can be explained by different mechanisms that are not exclusive but complementary. Screening by the dermatologist for MetS in patients with acne vulgaris could prove advantageous for the detection of at-risk individuals and the initiation of preventive therapy before cardiovascular disease or diabetes mellitus sets in.
